# Supplementary material for: Genome-wide quantitative dissection of an arthropod segmented body plan at single-cell resolution
Source: Commun Biol. 2025 Jun 11;8:913. doi: 10.1038/s42003-025-08335-x (PMC12159194; doi:10.1038/s42003-025-08335-x)
Supplement: Supplementary file 1 — Supplementary Information [file 42003_2025_8335_MOESM1_ESM.pdf]

Genome-wide quantitative dissection of an arthropod segmented body plan at single-cell resolution

Takanori Akaiwa[1,2, †], Hiroki Oda[1,2] & Yasuko Akiyama-Oda[1,3,4, *]

[1] JT Biohistory Research Hall, 1-1 Murasaki-cho, Takatsuki, Osaka 569-1125, Japan.
[2] Department of Biological Science, Graduate School of Science, Osaka University, Toyonaka, Osaka, Japan.
[3] Department of Microbiology and Infection Control, Faculty of Medicine, Osaka Medical and Pharmaceutical University, Takatsuki, Osaka, Japan.
[4] PRESTO, Japan Science and Technology Agency, Kawaguchi, Saitama, Japan.
[†]Present address: Exploratory Research Center on Life and Living Systems (ExCELLS), National Institutes of Natural Sciences, Okazaki, Aichi, Japan
*Corresponding author, email: yasuko@brh.co.jp

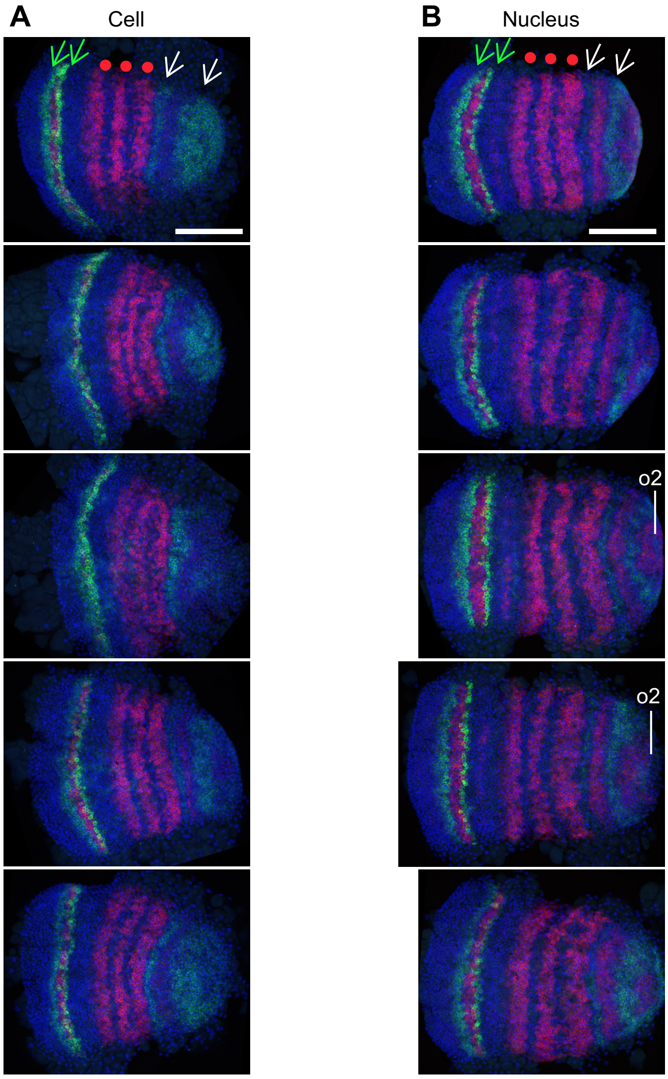

Supplementary Figure 1. Expression of *Pt-hh* and *Pt-msx1*. A, B Sibling embryos of those used for single-cell (A) and single-nucleus (B) library construction. Green arrows indicate bi-splitting *Pt-hh* stripes in the head, red dots indicate tri-splitting *Pt-msx1* stripes in the thorax, and white arrows point to L4 and O1 *Pt-hh* stripes formed by oscillation. Two embryos showed *Pt-hh* expression in the O2 segment. The embryo displayed at the top in B is the same as that shown in Fig. 5A. Scale bars = 200 µm.

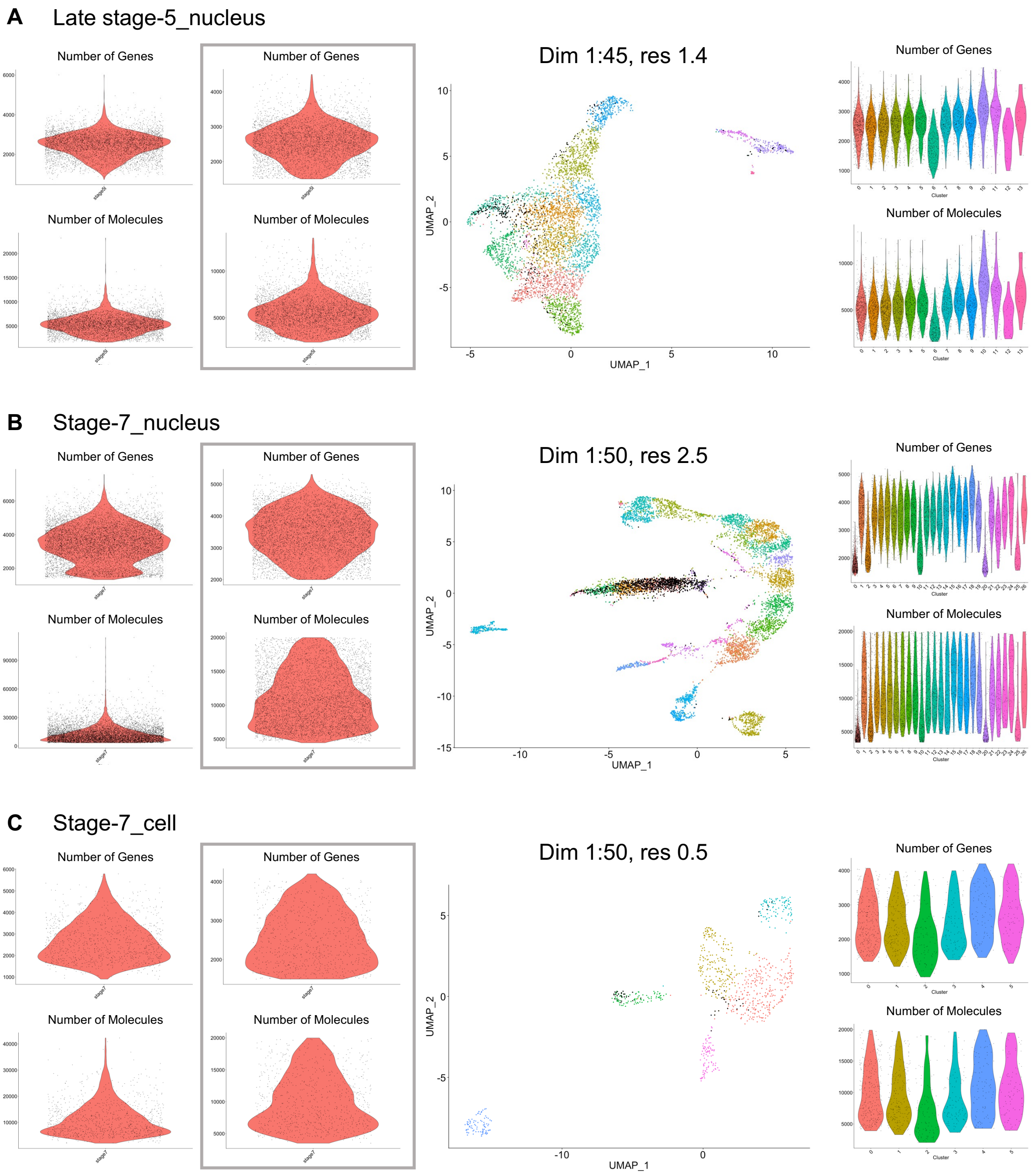

Supplementary Figure 2. Clustering analyses of the unfiltered cell/nuclei dataset. A–C Violin plots showing the number of genes (upper panels) and molecules (lower panels) detected in each cell or nucleus before (left column) and after (boxed in gray) filtering, UMAP plots showing results of the clustering of the unfiltered datasets (third column), and violin plots for the counts in each cluster shown in the UMAP plots (right) in the late stage-5_nucleus (A), stage-7_nucleus (B), and stage-7_cell (C) datasets. In the UMAP plots, the positions of nuclei or cells that were filtered out are visualized by black dots.

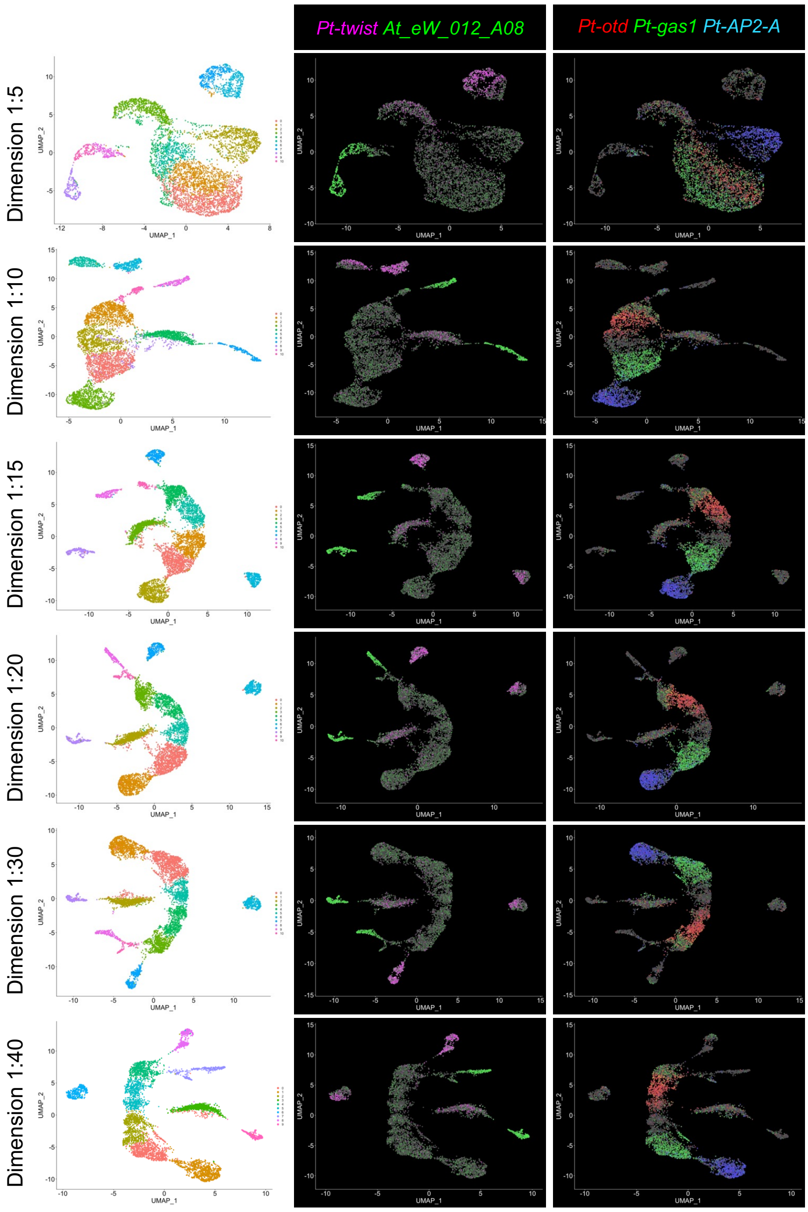

Supplementary Figure 3. UMAP plots of stage-7_nucleus RNA-seq data generated with multiple dimension parameters. Expression of the mesodermal marker *Pt-twist*, endodermal marker *At_eW_012_A08*, and body region markers *Pt-otd* (head), *Pt-gas1* (thorax), and *Pt-AP2A* (opisthosoma) is also shown.

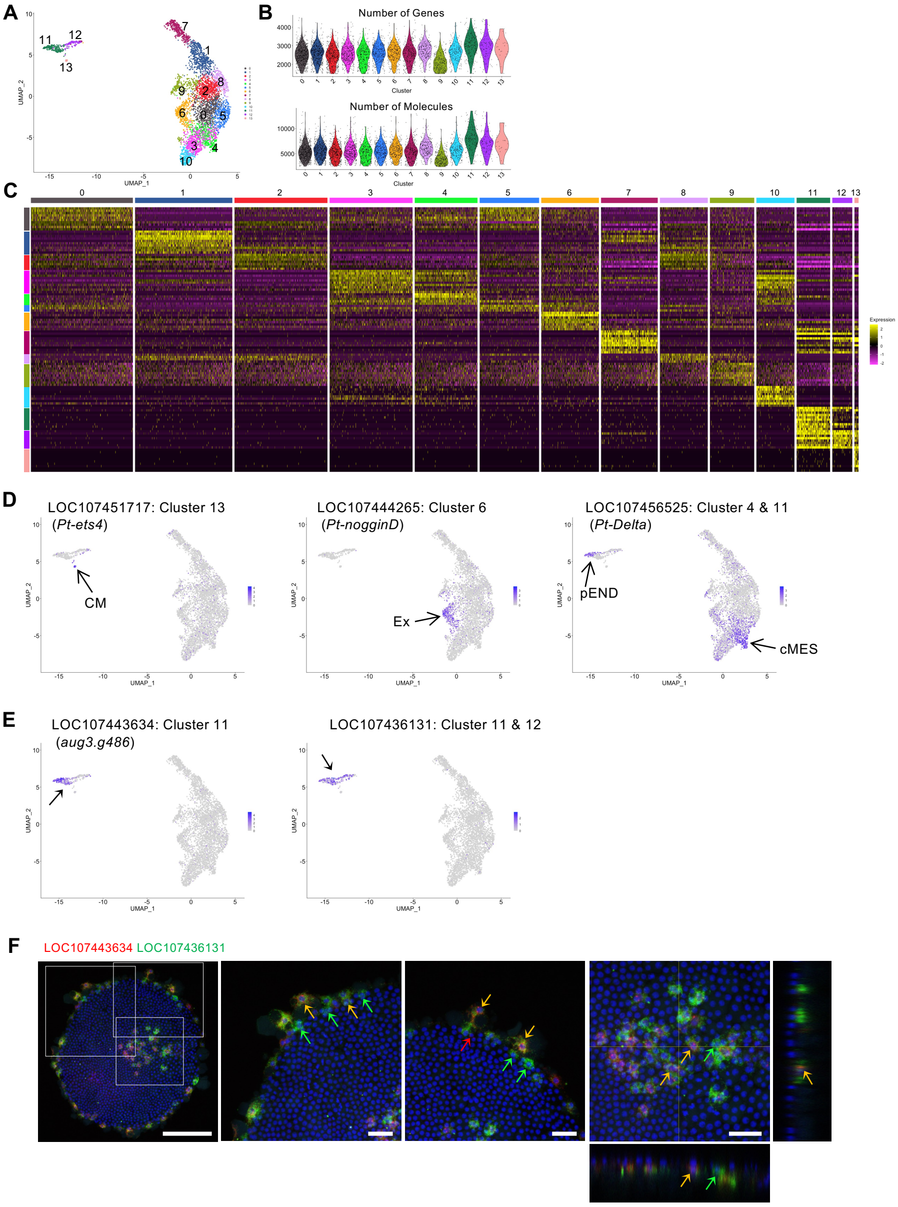

Supplementary Figure 4. Clustering of late stage-5_nucleus data and detection of endoderm clusters. A, B UMAP and violin plots showing clusters of late stage-5_nucleus RNA-seq data. These are the same as in Fig. 1C and F. **C** Heat map showing expression levels (log-normalized) of markers in nuclei of each cluster. Top-10 markers of each cluster are shown, but those that have already appeared in earlier clusters are not shown. **D** Expression of cell-type markers in the UMAP plot. LOC107451717 (*Pt-ets4*), the CM cells; LOC107444265 (*Pt-noggin-D*), extraembryonic cells; LOC107456525 (*Pt-Delta*), cMES and pEND cells. **E** Marker genes used for the analysis of clusters 11 and 12. Arrows show the position of expressing nuclei (D, E). **F** FISH of the markers in the late stage-5 embryo. LOC107443634 (*aug3.g486*) (red) and LOC107436131 (green). The embryo was counterstained with DAPI (blue). Boxed regions are magnified. Cross-section views along the lines are also shown (right panel). Green and red arrows indicate single-positive cells, whereas yellow arrows point to double-positive cells. Scale bars = 200 and 50 µm (magnified views).

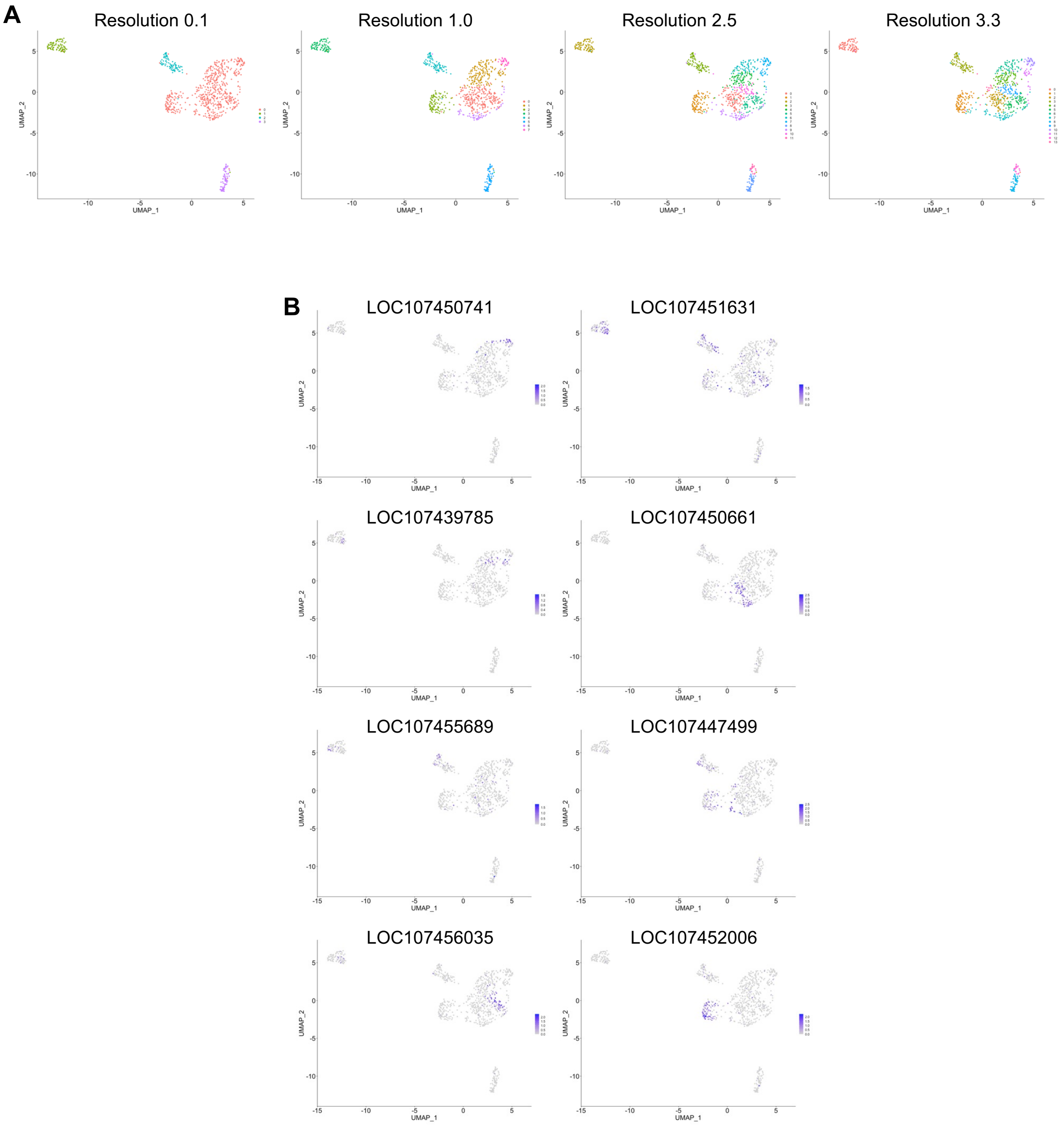

Supplementary Figure 5. Stage-7_cell UMAP plots. A UMAP plots generated with the resolution parameters, 0.1, 1.0, 2.5, and 3.3, at a dimension of 1:50. **B** Expression of AP markers. The same gene set as in Fig. 3A.

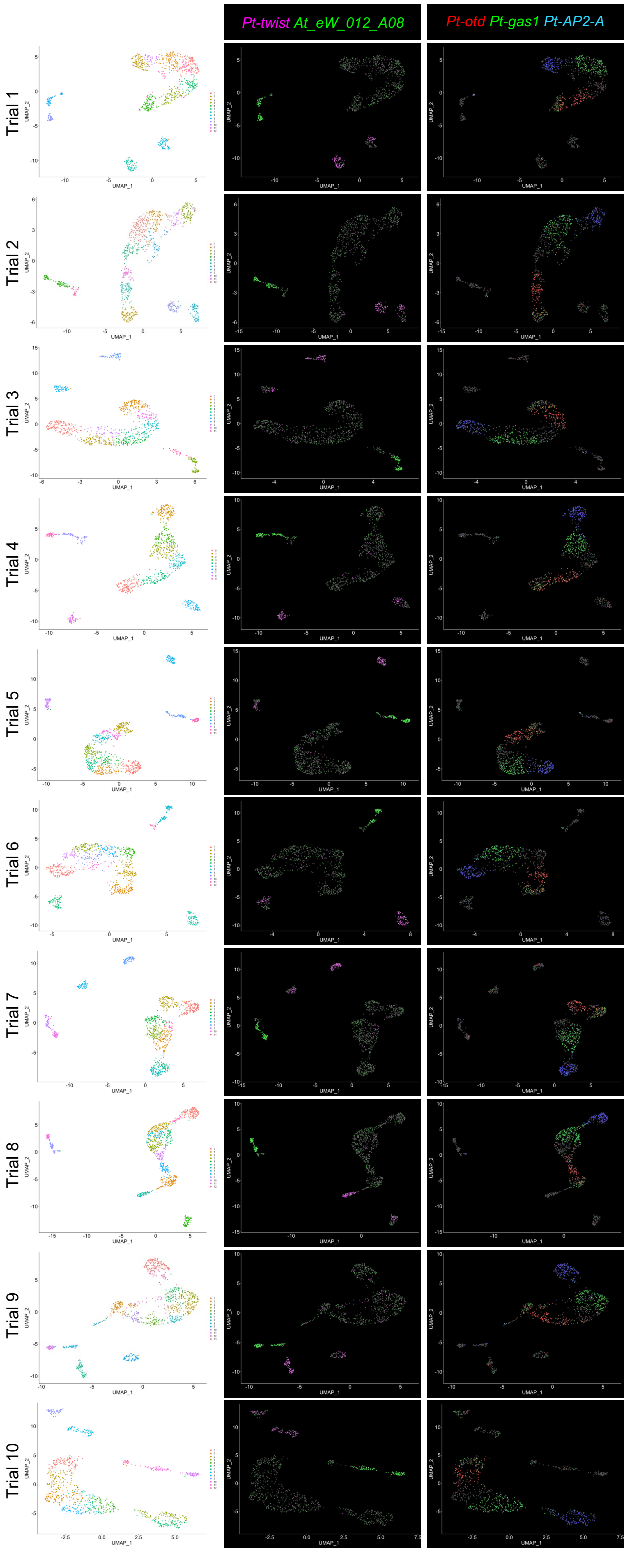

Supplementary Figure 6. Stage-7_nucleus UMAP plots generated with randomly selected 865 nuclei. Results of 10 trials are shown. Expression of the mesodermal marker *Pt-twist*, endodermal marker *At_eW_012_A08*, and body region markers *Pt-otd* (head), *Pt-gas1* (thorax), and *Pt-AP2A* (opisthosoma).

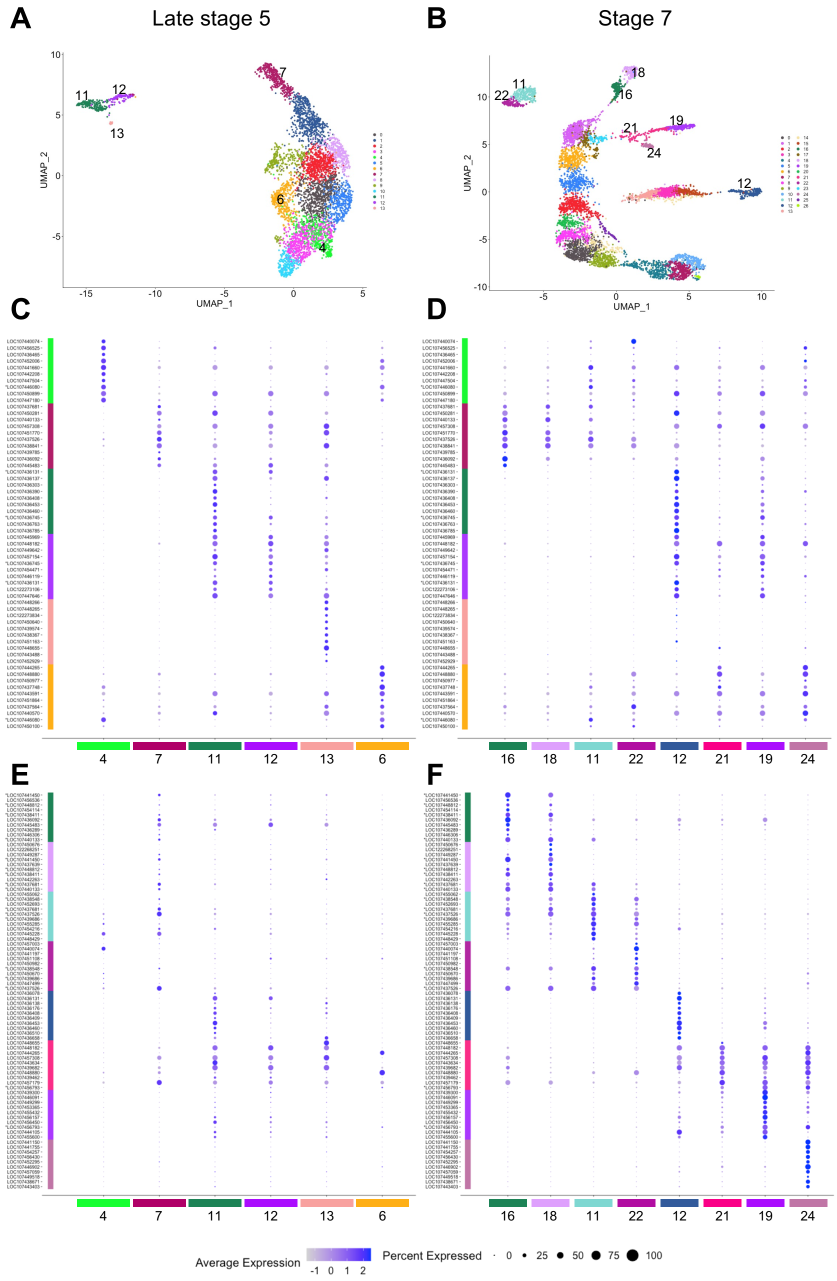

Supplementary Figure 7. Expression of mesoderm and endoderm cluster markers. A, B Clusters in the UMAP plots. Clusters of the mesoderm, endoderm, extraembryonic tissue, and CM cells are indicated by numbers. **C–F** Dot plots showing the expression of top-10 markers of the late stage-5 (C, D) and those of the stage-7 (E, F) clusters. Expression in the nuclei of the clusters at late stage 5 (C, E) and stage 7 (D, F) is shown. Average expression levels (log-normalized) and percent expressed in the nuclei in each cluster are shown by the colors and sizes of the circles. Asterisks indicate genes that appeared multiple times in the list (* twice, ** three times).

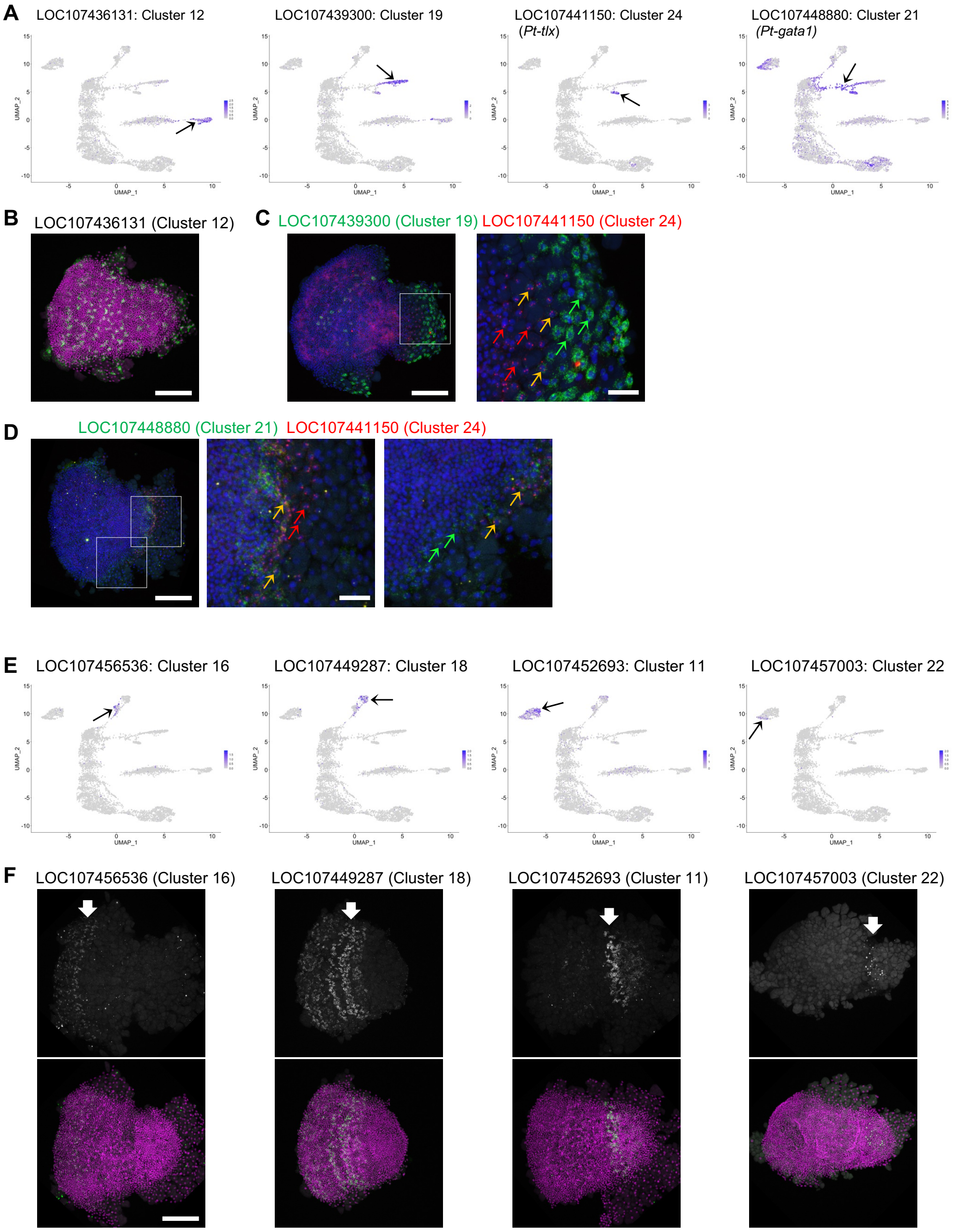

Supplementary Figure 8. Analysis of stage-7 endoderm and mesoderm clusters. A Marker genes used for the analysis of endoderm and extraembryonic clusters. Arrows show the positions of the clusters in the UMAP. **B–D** FISH images of stage-7 embryos. LOC107436131 (B, green), LOC107439300 (C, green), LOC107441150 (C, D, red), and LOC107448880 (D, green). Embryos were counterstained with DAPI (B, purple; C, D, blue). Boxed regions are magnified. Green and red arrows indicate single-positive cells, whereas yellow arrows point to double-positive cells. **E** Marker genes used for the analysis of mesoderm clusters. Arrows show the positions of the clusters in the UMAP. **F** FISH images of the mesoderm markers in the stage-7 germ band. Signals are shown in upper and lower (green) panels. Embryos were counterstained with DAPI (lower panels, purple). White arrows show expression domains. Scale bars = 200 and 50 µm (magnified views).

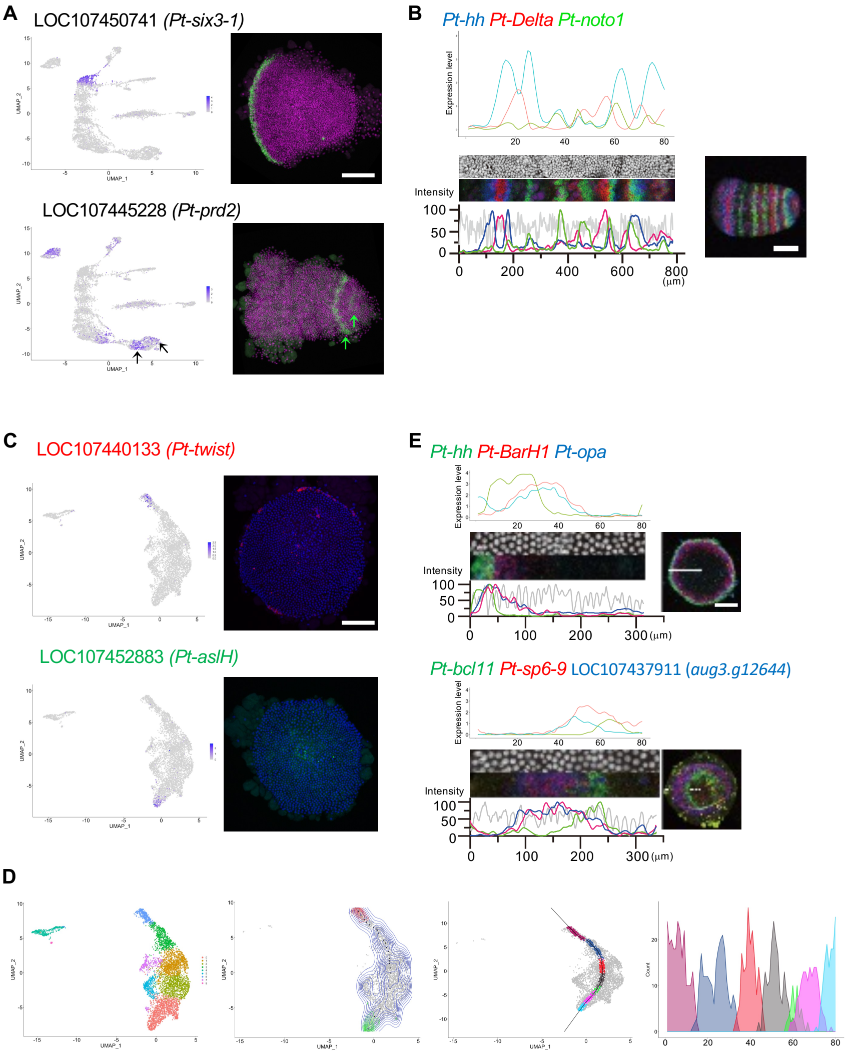

Supplementary Figure 9. Generation of expression profiles and comparison with embryo staining. A Expression of LOC107450741 (*Pt-six3-1*) and LOC107445228 (*Pt-prd2*) in the stage-7_nucleus UMAP and stage-7 embryos. These genes were used for generation of the contours. *Pt-prd2* stripes in the opisthosoma are indicated by arrows. **B** Comparison of expression profiles of *Pt-hh*, *Pt-Delta*, and *Pt-noto1* (top) with the signal intensities along the midline of stage-7 embryos stained for transcripts of these genes (bottom). **C** Expression of LOC107440133 (*Pt-twist*) and LOC107452883 (*Pt-aslH*) in the late stage-5_nucleus UMAP and late stage-5 embryos. **D** Process for generation of the expression profile. Left-to-right: A UMAP plot generated with a dimension 1:45 and resolution 0.5, contours based on the densities of ectoderm, mesoderm, and extraembryonic nuclei (blue) and of those expressing *Pt-twist* (red) and *Pt-aslH* (green) and points plotted along the ridge, the spline curve, and the distribution of the nuclei along the linear axis. The color code corresponds to the cluster colors (Fig. 1C). **E** Comparison of expression profiles of genes with the signal intensities along the radius of the late stage-5 germ discs stained for transcripts of the same genes. The examined genes were *Pt-hh*, *Pt-BarH1*, and *Pt-opa*, and *Pt-bcl11*, *Pt-sp6-9*, and *aug3.g12644* (LOC107437911). Scale bars = 200 µm. Photos of embryos and graphs of intensities in B and E are from Akiyama-Oda and Oda[38] (CCBY4.0).

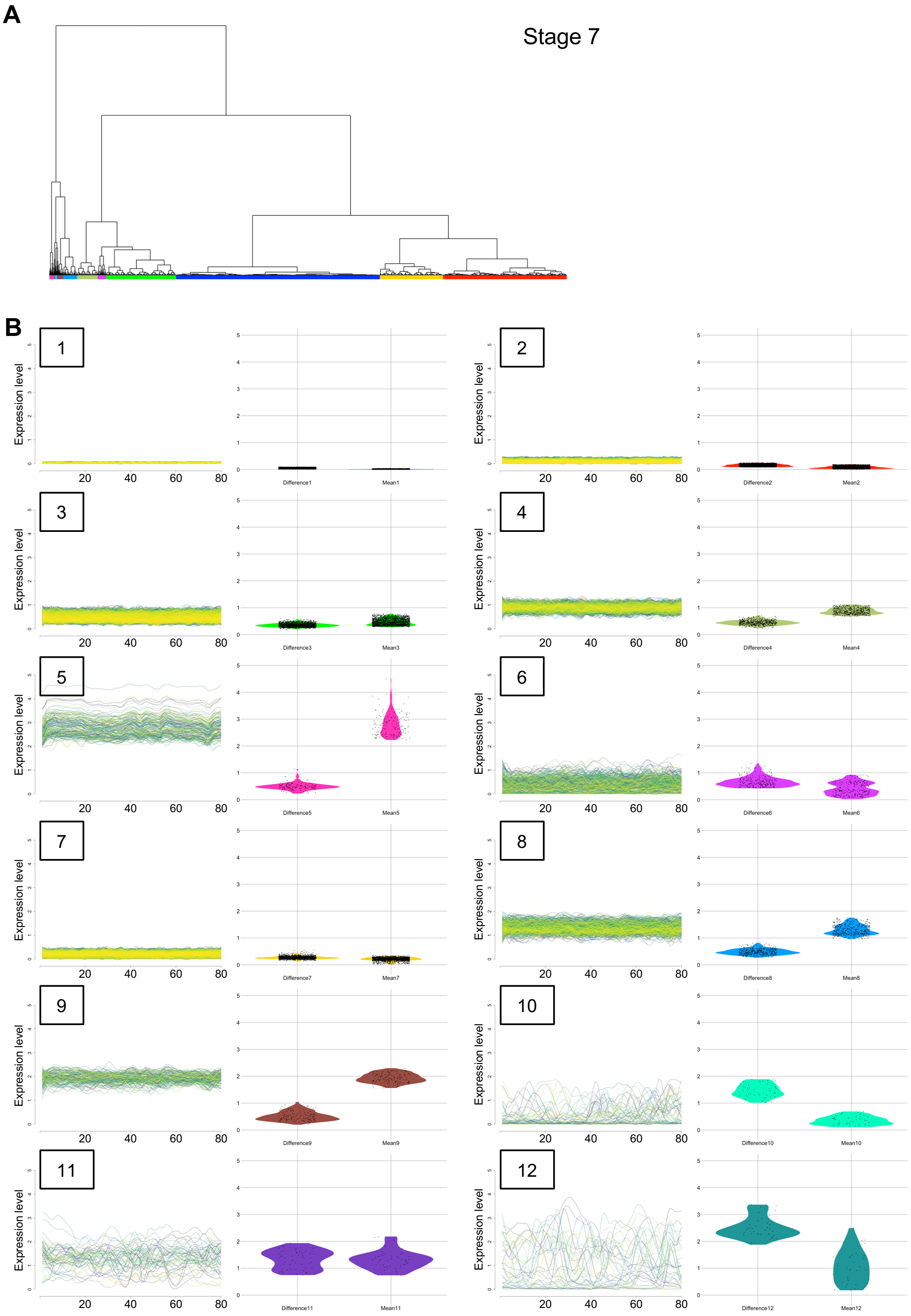

Supplementary Figure 10. Hierarchical clustering of stage-7 genes. A Dendrogram of genes generated with the mean and difference between the maximum and minimum values of the expression profiles. **B** Expression profiles of 12 groups of genes generated in the first round of clustering. Violin plots on the right show the difference and mean values of genes included in the clusters. Colors of the violin plots correspond to the colors at the bottom of the dendrogram (A).

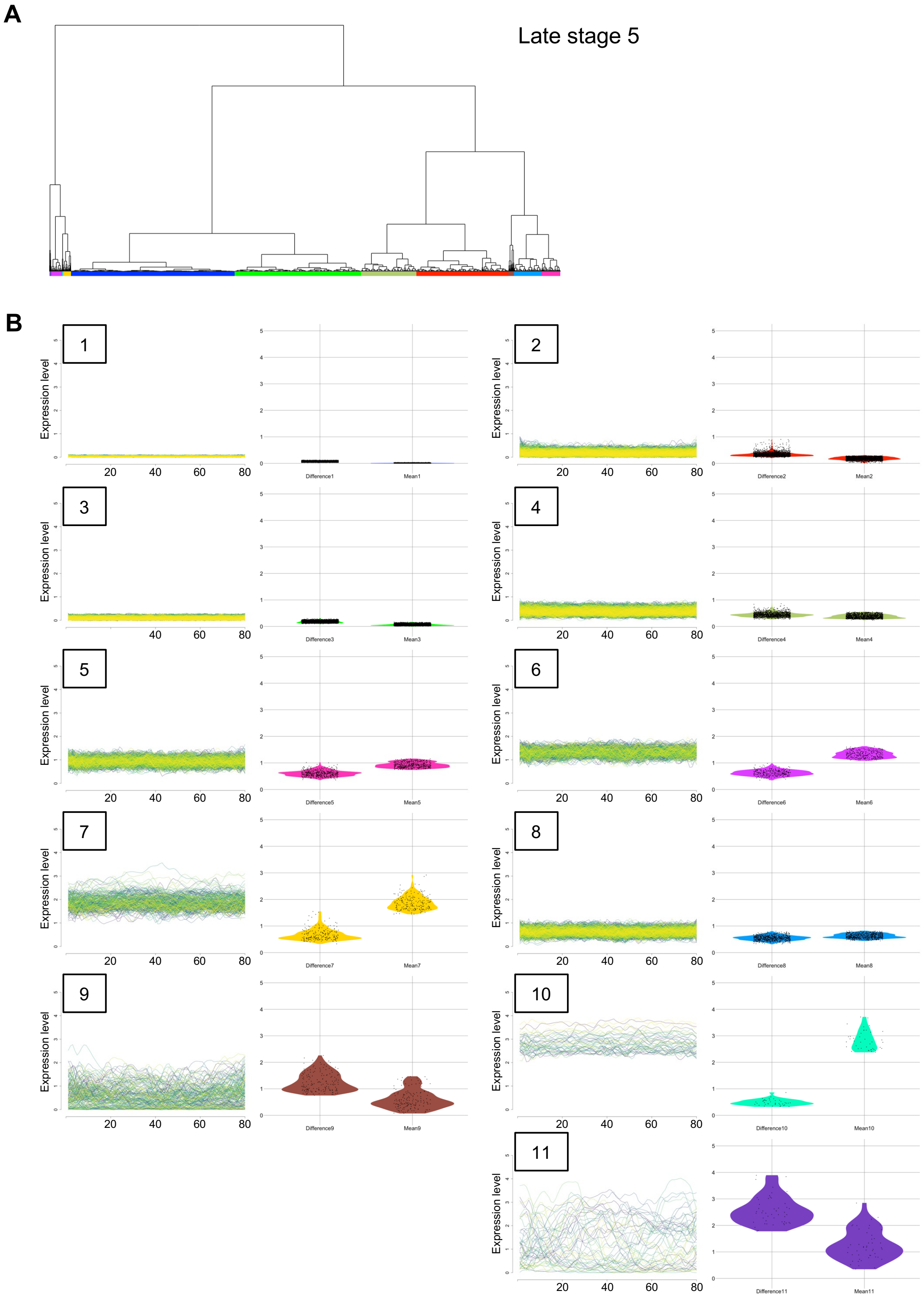

A
Late stage 5
B
1
2
3
4
5
6
7
8
9
10
11
Expression level
20
40
60
80
Difference1
Mean1
Difference2
Mean2
Difference3
Mean3
Difference4
Mean4
Difference5
Mean5
Difference6
Mean6
Difference7
Mean7
Difference8
Mean8
Difference9
Mean9
Difference10
Mean10
Difference11
Mean11

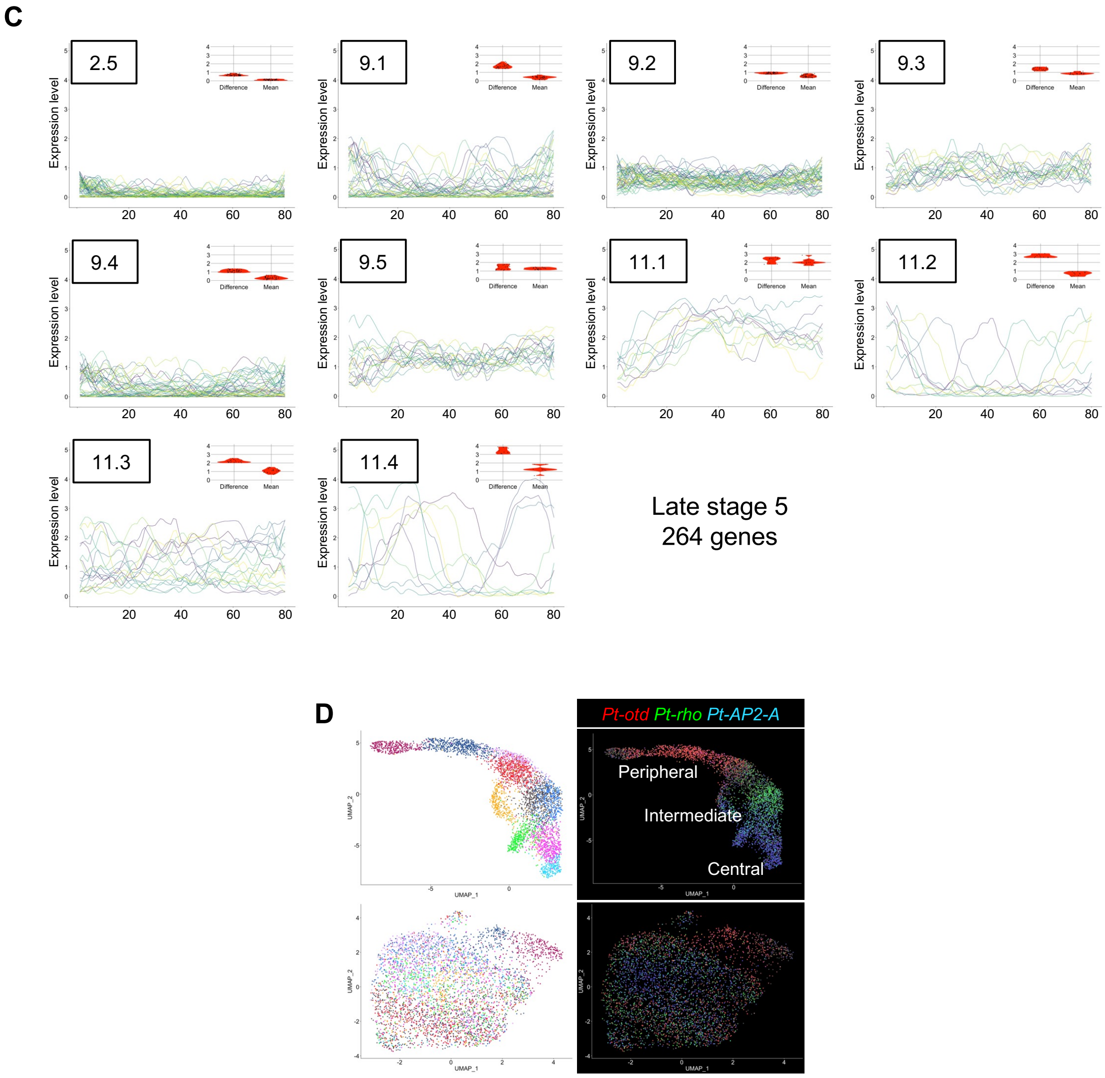

Supplementary Figure 11. Hierarchical clustering of late stage-5 genes. A Dendrogram of genes generated with the mean and difference between the maximum and minimum values of the expression profiles. **B** Expression profiles of 11 groups of genes generated in the first round of clustering. Violin plots on the right show the difference and mean values of genes included in the clusters. Colors of the violin plots correspond to the colors at the bottom of the dendrogram (A). **C** Expression profiles of 264 selected genes visualized separately by subgroups. Insets show violin plots of the difference and mean values of genes included in the subclusters. **D** UMAP plots generated with only the 264 selected genes (top) and without the 264 genes (bottom). Expression patterns of body region markers *Pt-otd*, *Pt-rho*, and *Pt-AP2A* are shown.

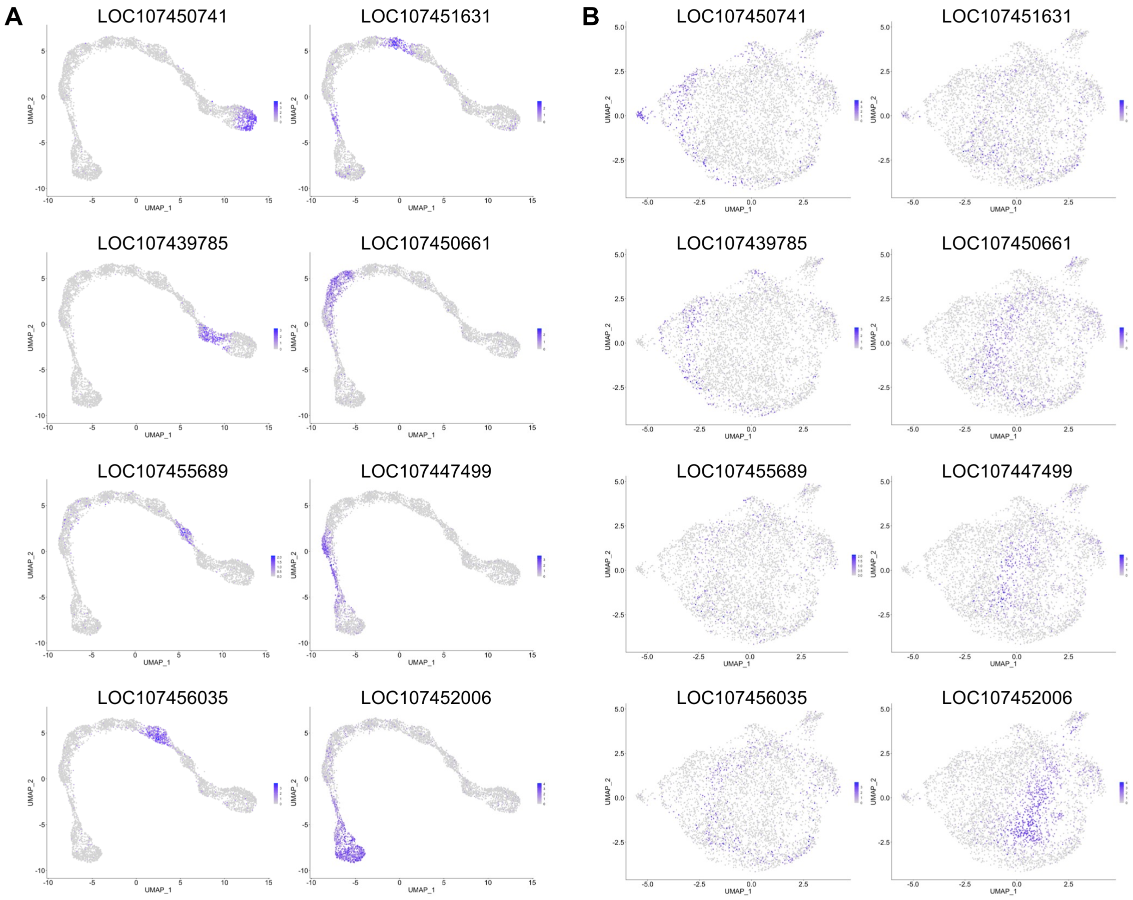

Supplementary Figure 12. Expression of AP marker genes in UMAP plots. A, B UMAP plots generated with submatrices A (A) and B (B) from the full matrix of stage-7_nucleus. The genes presented are the same as those in Fig. 3A.

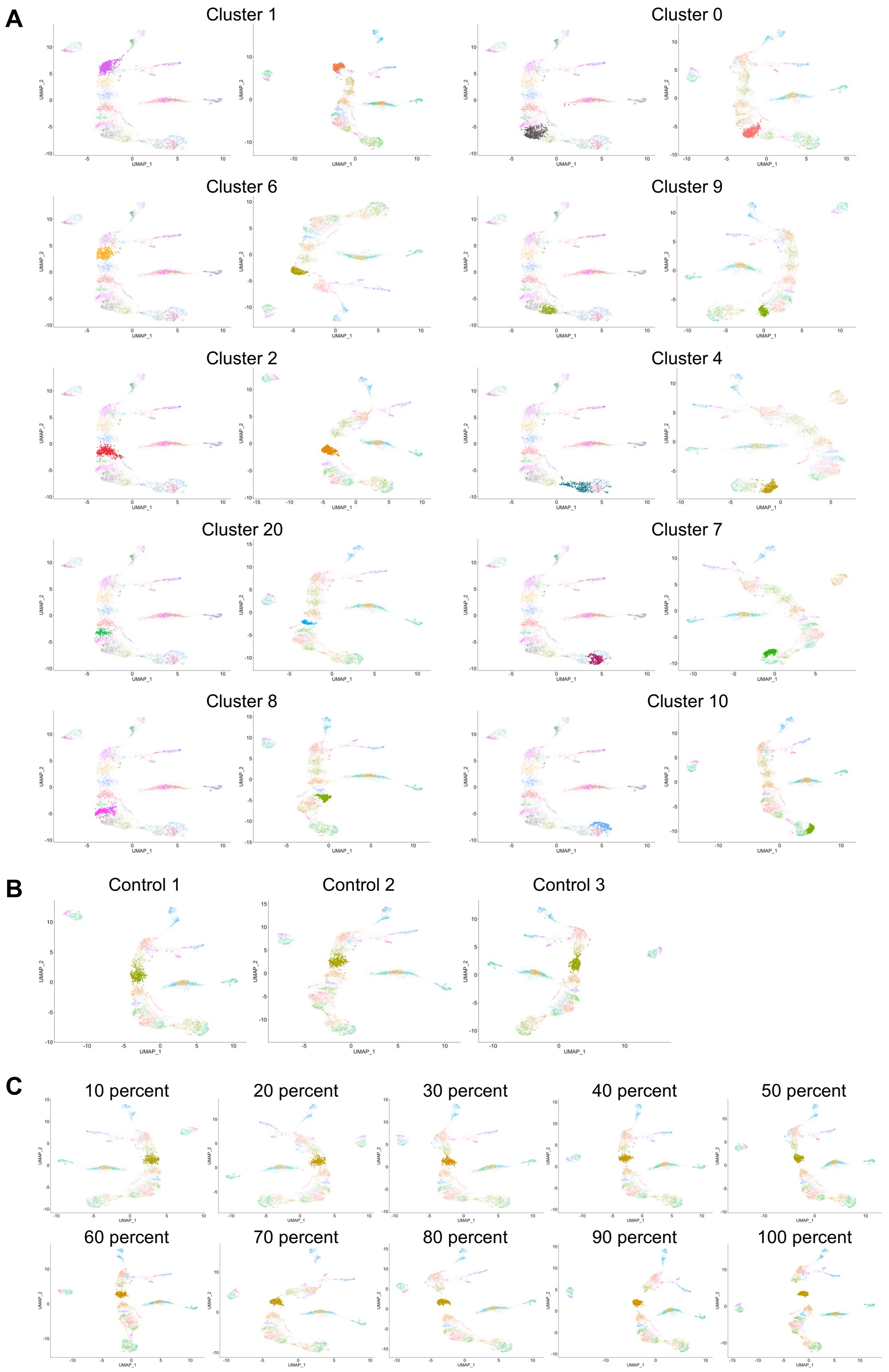

Supplementary Figure 13. Randomization experiments of the 213 genes. A Randomization of the expression of the 213 selected genes in the nuclei of each cluster. The stage-7_nucleus UMAP plots showing nuclei of a cluster without (left) and with randomization of the 213 selected genes (right). **B** UMAP plots showing results of randomization of other 213 genes in the nuclei of cluster 5. Three trials are shown. Control 1 is the same as shown in Fig. 6G. **C** Randomization of the expression of the 213 selected genes in a part of the nuclei in cluster 5. Randomization was done in 10–100% of the cluster-5 nuclei.

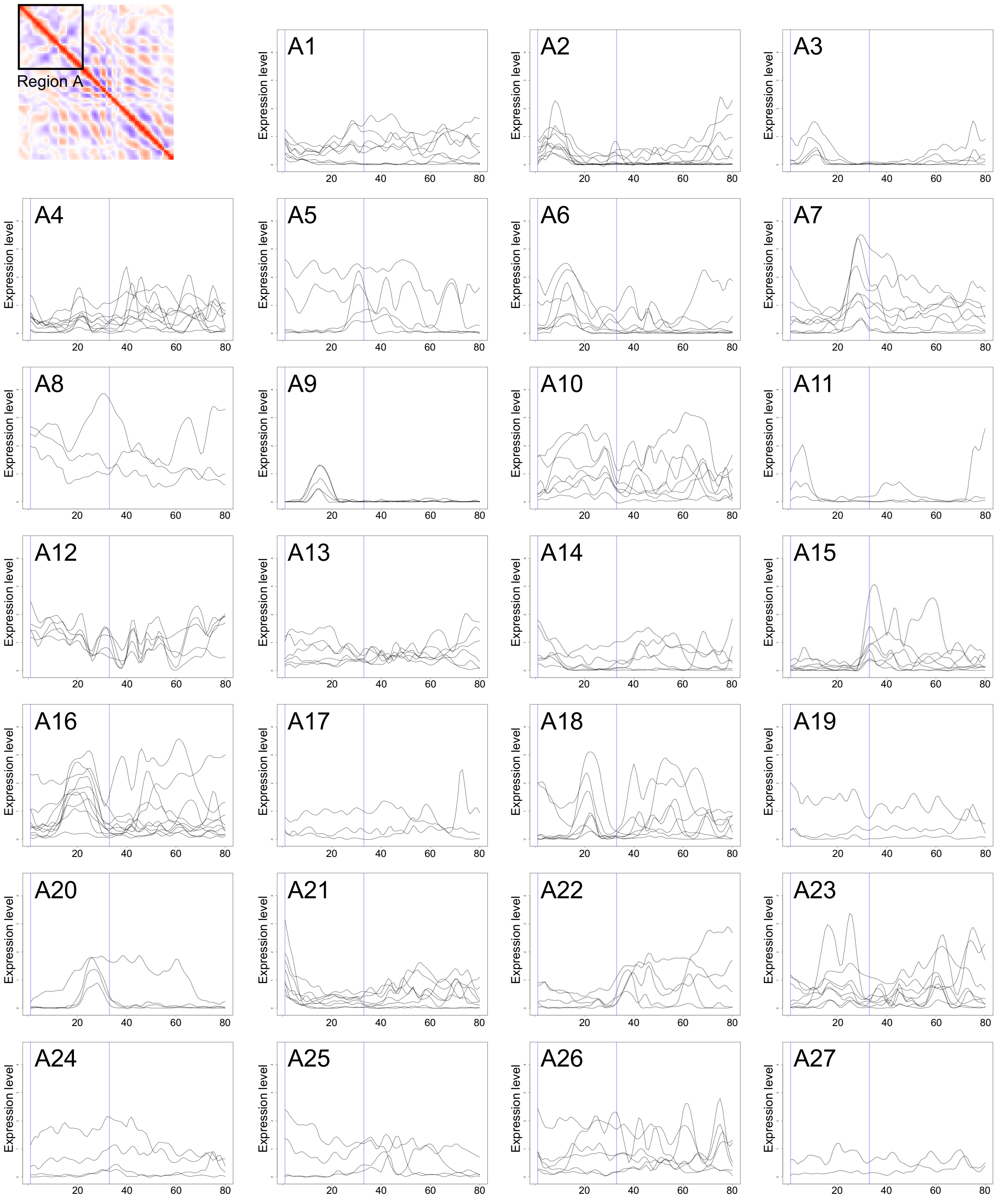

Supplementary Figure 14. Grouping of the 213 selected genes using the stage-7 first-derivative values of the expression profiles in region A.

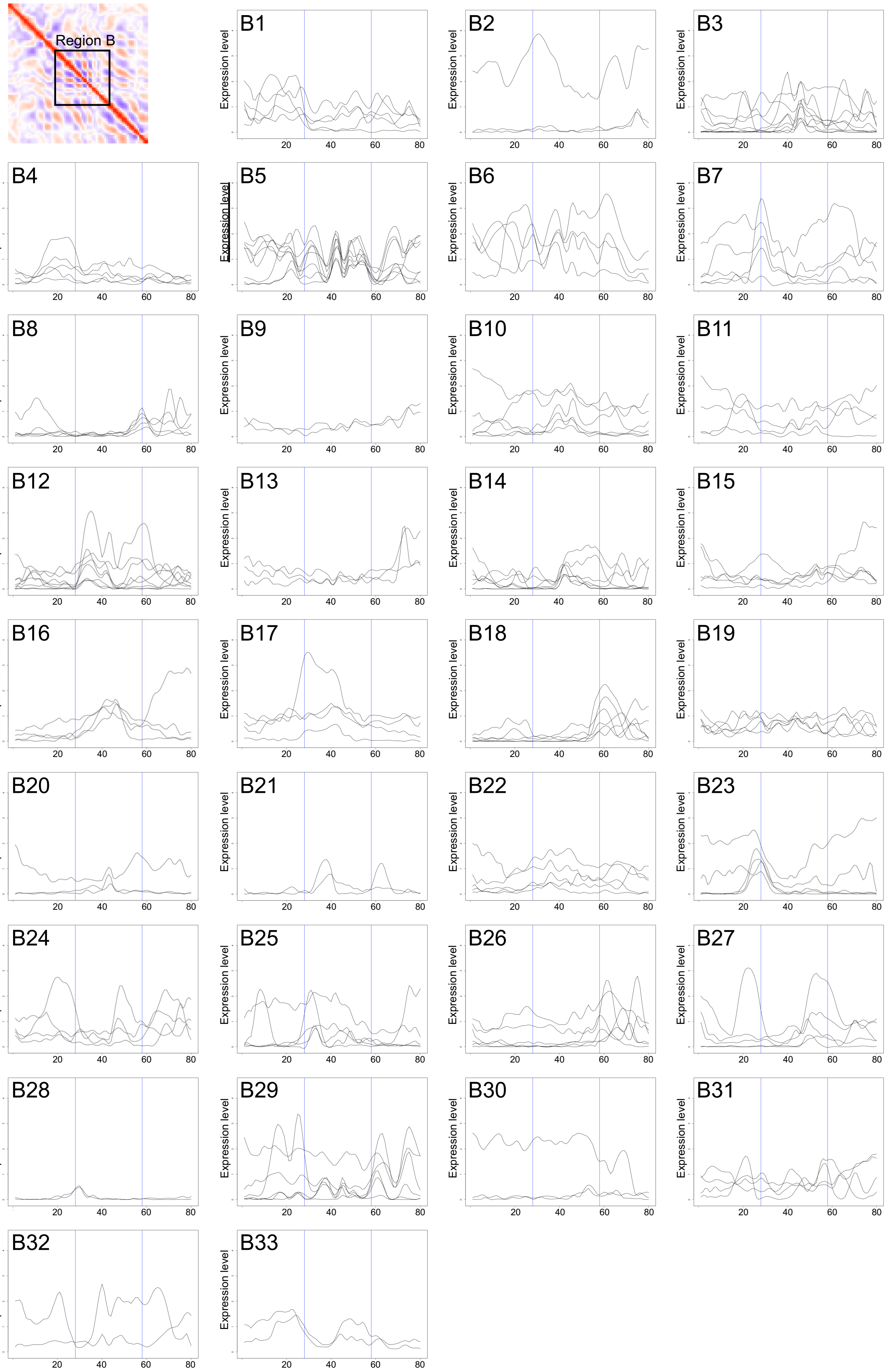
Region B
B1
B2
B3
B4
B5
B6
B7
B8
B9
B10
B11
B12
B13
B14
B15
B16
B17
B18
B19
B20
B21
B22
B23
B24
B25
B26
B27
B28
B29
B30
B31
B32
B33
Expression level
20
40
60
80

Supplementary Figure 15. Grouping of the 213 selected genes using the stage-7 first-derivative values of the expression profiles in region B.

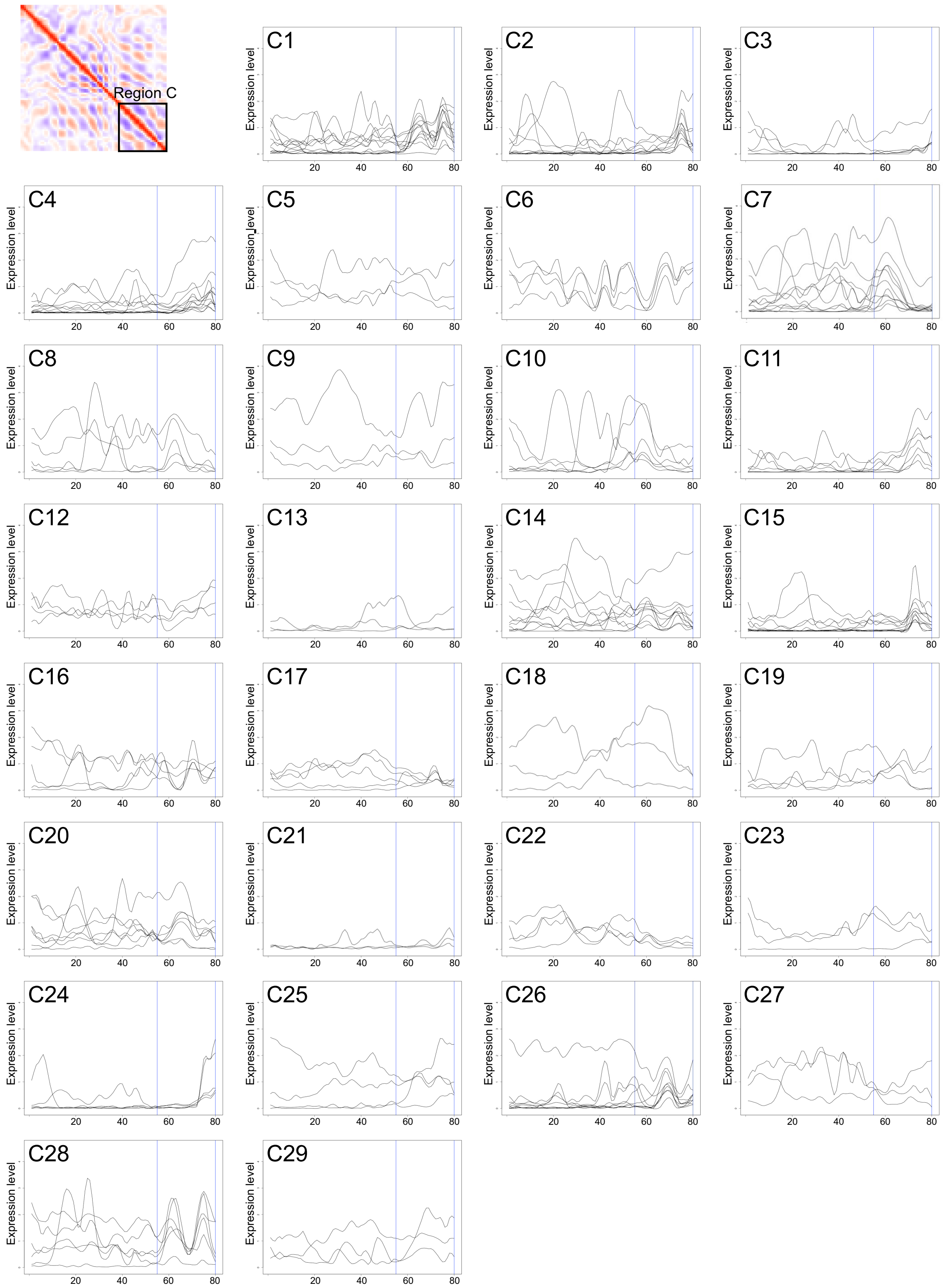

Supplementary Figure 16. Grouping of the 213 selected genes using the stage-7 first-derivative values of the expression profiles in region C.

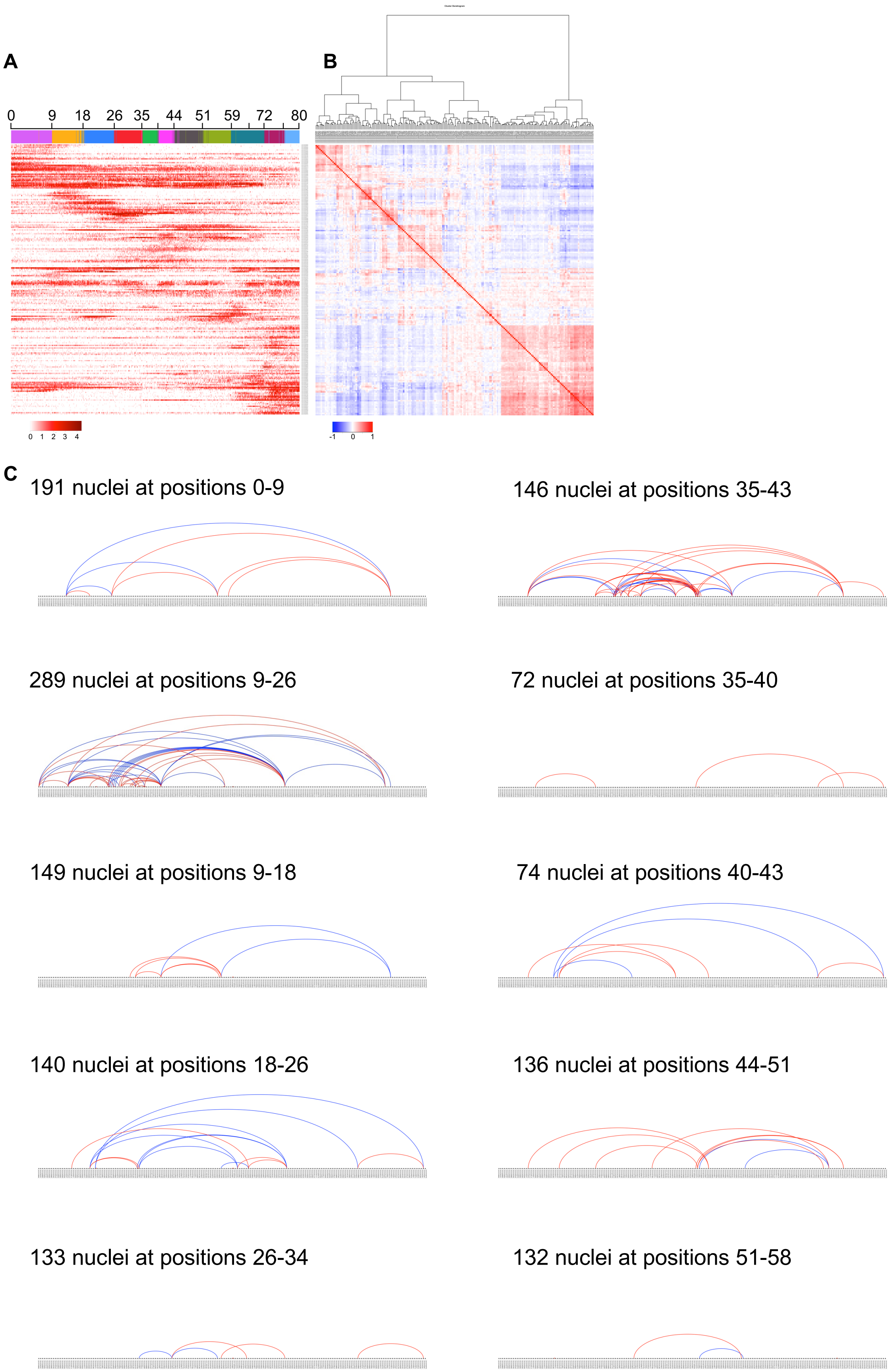
A
B
0
9
18
26
35
44
51
59
72
80
0 1 2 3 4
-1 0 1
C
191 nuclei at positions 0-9
146 nuclei at positions 35-43
289 nuclei at positions 9-26
72 nuclei at positions 35-40
149 nuclei at positions 9-18
74 nuclei at positions 40-43
140 nuclei at positions 18-26
136 nuclei at positions 44-51
133 nuclei at positions 26-34
132 nuclei at positions 51-58

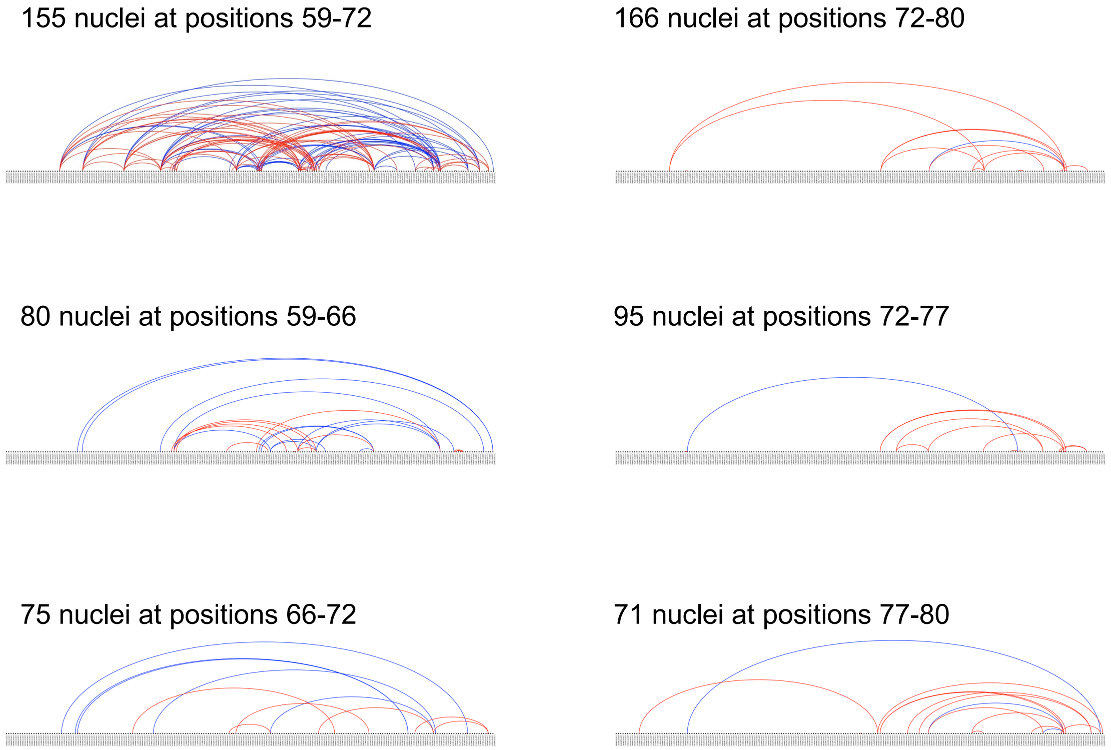

Supplementary Figure 17. Correlation coefficients between the 213 selected genes at stage 7. A Heat map showing the normalized expression levels of the 213 genes in single nuclei aligned by the position along the reconstructed axis. Only the colored nuclei in Fig. 5E are shown. The color bar at the top indicates the cluster colors in Fig. 2. **B** Dendrogram and 2-D plot of correlation coefficients for the 213 genes, calculated using the expression levels in all the colored nuclei. **C** Highly correlated pairs among the 213 genes. Correlation coefficients between the genes were calculated with the expression levels in the nuclei located at the indicated positions. Gene pairs with $r > 0.5$ and $r < -0.5$ are connected with red and blue lines, respectively. The alignments of the genes in A (row), B (row and the dendrogram), and C are the same.
