# Supplementary material for: Genome-wide quantitative dissection of an arthropod segmented body plan at single-cell resolution
Source: Commun Biol. 2025 Jun 11;8:913. doi: 10.1038/s42003-025-08335-x (PMC12159194; doi:10.1038/s42003-025-08335-x)
Supplement: Supplementary file 2 — Description of Additional Supplementary Files [file 42003_2025_8335_MOESM2_ESM.docx]

Description of Additional Supplementary Files

**File name:** Supplementary Data 1

Statistics of stage7_single-cell/nucleus RNA-seq experiments.

**File name:** Supplementary Data 2

**Description:** Stage-7_nucleus cluster markers.

**File name:** Supplementary Data 3

**Description:** Late stage-5_nucleus cluster markers.

**File name:** Supplementary Data 4

**Description:** Positions of ectoderm nuclei in the stage-7_nucleus UMAP.

**File name:** Supplementary Data 5

**Description:** Gene expression profiles at stage 7.

**File name:** Supplementary Data 6

**Description:** Positions of ectoderm and mesoderm nuclei in the late stage-5_nucleus UMAP.

**File name:** Supplementary Data 7

**Description:** Gene expression profiles at late stage 5.

**File name:** Supplementary Data 8

**Description:** Results of hierarchical clustering and used values from the stage-7 expression profiles.

**File name:** Supplementary Data 9

**Description:** 213 genes selected from the expression profiles at stage 7.

**File name:** Supplementary Data 10

**Description:** Results of hierarchical clustering and used values from the late stage-5 expression profiles.

**File name:** Supplementary Data 11

**Description:** 264 genes selected from the expression profiles at late stage 5.

**File name:** Supplementary Data 12

**Description:** First derivative values of 213 genes at 80 positions of the stage-7 expression profiles.

**File name:** Supplementary Data 13

**Description:** First derivative values of 264 genes at 80 positions of the late stage-5 expression profiles.

**File name:** Supplementary Data 14

**Description:** List of gene groups generated using the first derivative values at stage 7.

**File name:** Supplementary Data 15

**Description:** Gene pairs with high correlation coefficient calculated using the expression levels at single nuclei in each region.

**File name:** Supplementary Data 16

**Description:** List of genes excluded from the hierarchical clustering analysis of the stage-7_nucleus data.

**File name:** Supplementary Data 17

**Description:** List of genes excluded from the hierarchical clustering analysis of the late stage-5_nucleus data.

**File name:** Supplementary Data 18

**Description:** Clones.
