# Supplementary material for: Genome-wide quantitative dissection of an arthropod segmented body plan at single-cell resolution
Source: Commun Biol. 2025 Jun 11;8:913. doi: 10.1038/s42003-025-08335-x (PMC12159194; doi:10.1038/s42003-025-08335-x)
Supplement: Supplementary file 21 — reporting summary [file 42003_2025_8335_MOESM21_ESM.pdf]

nature portfolio

Corresponding author(s): Yasuko Akiyama-Oda

Last updated by author(s): May 26, 2025

Reporting Summary

Nature Portfolio wishes to improve the reproducibility of the work that we publish. This form provides structure for consistency and transparency in reporting. For further information on Nature Portfolio policies, see our Editorial Policies and the Editorial Policy Checklist.

Statistics

For all statistical analyses, confirm that the following items are present in the figure legend, table legend, main text, or Methods section.

n/a	Confirmed	
☐	☒	The exact sample size (*n*) for each experimental group/condition, given as a discrete number and unit of measurement
☐	☒	A statement on whether measurements were taken from distinct samples or whether the same sample was measured repeatedly
☐	☒	The statistical test(s) used AND whether they are one- or two-sided<br>*Only common tests should be described solely by name; describe more complex techniques in the Methods section.*
☒	☐	A description of all covariates tested
☐	☒	A description of any assumptions or corrections, such as tests of normality and adjustment for multiple comparisons
☐	☒	A full description of the statistical parameters including central tendency (e.g. means) or other basic estimates (e.g. regression coefficient) AND variation (e.g. standard deviation) or associated estimates of uncertainty (e.g. confidence intervals)
☐	☒	For null hypothesis testing, the test statistic (e.g. *F*, *t*, *r*) with confidence intervals, effect sizes, degrees of freedom and *P* value noted<br>*Give P values as exact values whenever suitable.*
☒	☐	For Bayesian analysis, information on the choice of priors and Markov chain Monte Carlo settings
☒	☐	For hierarchical and complex designs, identification of the appropriate level for tests and full reporting of outcomes
☒	☐	Estimates of effect sizes (e.g. Cohen's *d*, Pearson's *r*), indicating how they were calculated

Our web collection on statistics for biologists contains articles on many of the points above.

Software and code

Policy information about availability of computer code

Data collection	Single-cell and single-nucleus libraries were analyzed using HiSeqX Sequencing system (illumina). Images were collected using a TCS SPE confocal system (Leica).
Data analysis	For single-cell RNA-seq and single-nucleus RNA-seq analysis, paired-end fastq data were processed using the BD Rhapsody WTA pipeline on the Seven Bridges Genomics Cloud platform with default parameters. The R2 reads were aligned to the Parasteatoda tepidariorum genome, GCF_000365465.3_Ptep_3.0. For single-nucleus RNA-seq data, the annotation file was modified to include both exonic and intronic reads for read counting. The resulting datasets were analyzed using the Seurat package (v.4.1.1). Additional custom analyses were performed using R and python scripts. R functions and packages used include sample(), geom_density_2d() (ggplot2), xspline(), geom_smooth() (ggplot2), and cor(). The scripts used for analyzing the data and figure generation are available at https://github.com/Takanori-Akaiwa. Image analysis was performed using Fiji (ImageJ).

For manuscripts utilizing custom algorithms or software that are central to the research but not yet described in published literature, software must be made available to editors and reviewers. We strongly encourage code deposition in a community repository (e.g. GitHub). See the Nature Portfolio guidelines for submitting code & software for further information.

Data

Policy information about availability of data

All manuscripts must include a data availability statement. This statement should provide the following information, where applicable:

- Accession codes, unique identifiers, or web links for publicly available datasets
- A description of any restrictions on data availability
- For clinical datasets or third party data, please ensure that the statement adheres to our policy

Sequencing data generated in this study have been deposited in the NCBI Gene Expression Omnibus, under accession number GSE287446. Both row and processed expression matrices are available under the same accession. Gene expression patterns in the UMAP and patterns of the expression profiles have been deposited in figshare under accession number 10.6084/m9.figshare.c.7626428.

Research involving human participants, their data, or biological material

Policy information about studies with human participants or human data. See also policy information about sex, gender (identity/presentation), and sexual orientation and race, ethnicity and racism.

Reporting on sex and gender	n/a
Reporting on race, ethnicity, or other socially relevant groupings	n/a
Population characteristics	n/a
Recruitment	n/a
Ethics oversight	n/a

Note that full information on the approval of the study protocol must also be provided in the manuscript.

Field-specific reporting

Please select the one below that is the best fit for your research. If you are not sure, read the appropriate sections before making your selection.

☒ Life sciences ☐ Behavioural & social sciences ☐ Ecological, evolutionary & environmental sciences

For a reference copy of the document with all sections, see nature.com/documents/nr-reporting-summary-flat.pdf

Life sciences study design

All studies must disclose on these points even when the disclosure is negative.

Sample size	We did not perform sample size calculation for single-cell and single-nucleus RNA-sequencing . However, for the single-nucleus RNA-sequencing we examined data from more than 1.7 times the number of nuclei in a single stage-7 embryo and over twice the number of nuclei in a single late stage-5 embryo.
Data exclusions	For stage-7 single-cell data, only cells with expression of more than 1,500 unique genes and fewer than 20,000 total RNA molecules were retained. For stage-7 single-nucleus data, only nuclei with expression of more than 2,000 unique genes and fewer than 20,000 total RNA molecules were examined. For late stage-5 single-nucleus data, only nuclei with expression of more than 1,500 unique genes and fewer than 15,000 total RNA molecules were retained.
Replication	n/a
Randomization	Embryos showing typical morphological appearance were randomly selected.
Blinding	Blinding was not used in this study as single-cell and single-nucleus RNA-seq were not affected by investigators' expectations.

Reporting for specific materials, systems and methods

We require information from authors about some types of materials, experimental systems and methods used in many studies. Here, indicate whether each material, system or method listed is relevant to your study. If you are not sure if a list item applies to your research, read the appropriate section before selecting a response.

Materials & experimental systems

n/a	Involved in the study
☒	☐ Antibodies
☒	☐ Eukaryotic cell lines
☒	☐ Palaeontology and archaeology
☐	☒ Animals and other organisms
☒	☐ Clinical data
☒	☐ Dual use research of concern
☒	☐ Plants

Methods

n/a	Involved in the study
☒	☐ ChIP-seq
☒	☐ Flow cytometry
☒	☐ MRI-based neuroimaging

Animals and other research organisms

Policy information about studies involving animals; ARRIVE guidelines recommended for reporting animal research, and Sex and Gender in Research

Laboratory animals	Wild-type common house spider Parasteatoda tepidariorum maintained in the laboratory over 40 generations.
Wild animals	n/a
Reporting on sex	Sex of embryos was not considered in study design
Field-collected samples	n/a
Ethics oversight	All protocols and procedures were approved by the Animal Care and Use Committee of JT Biohistory Research Hall (2020-1-C1, 29-1, 40-1).

Note that full information on the approval of the study protocol must also be provided in the manuscript.

Plants

Seed stocks	*Report on the source of all seed stocks or other plant material used. If applicable, state the seed stock centre and catalogue number. If plant specimens were collected from the field, describe the collection location, date and sampling procedures.*
Novel plant genotypes	*Describe the methods by which all novel plant genotypes were produced. This includes those generated by transgenic approaches, gene editing, chemical/radiation-based mutagenesis and hybridization. For transgenic lines, describe the transformation method, the number of independent lines analyzed and the generation upon which experiments were performed. For gene-edited lines, describe the editor used, the endogenous sequence targeted for editing, the targeting guide RNA sequence (if applicable) and how the editor was applied.*
Authentication	*Describe any authentication procedures for each seed stock used or novel genotype generated. Describe any experiments used to assess the effect of a mutation and, where applicable, how potential secondary effects (e.g. second site T-DNA insertions, mosiacism, off-target gene editing) were examined.*
